# Meditation or Medication? Mindfulness training versus medication in the treatment of childhood ADHD: a randomized controlled trial

**DOI:** 10.1186/s12888-016-0978-3

**Published:** 2016-07-26

**Authors:** Renée Meppelink, Esther I. de Bruin, Susan M. Bögels

**Affiliations:** 1 Research Institute of Child Development and Education, University of Amsterdam, Nieuwe Achtergracht 127, 1018 WS Amsterdam, The Netherlands; 2 Research Priority Area Yield, University of Amsterdam, Amsterdam, The Netherlands; 3 UvA Minds, Academic outpatient child and adolescent treatment center of the University of Amsterdam, Amsterdam, The Netherlands

**Keywords:** Childhood ADHD, Mindfulness, Methylphenidate, RCT

## Abstract

**Background:**

Attention-Deficit-Hyperactivity-Disorder (ADHD) is, with a prevalence of 5 %, a highly common childhood disorder, and has severe impact on the lives of youngsters and their families. Medication is often the treatment of choice, as it currently is most effective. However, medication has only short-term effects, treatment adherence is often low and most importantly; medication has serious side effects. Therefore, there is a need for other interventions for youngsters with ADHD. Mindfulness training is emerging as a potentially effective training for children and adolescents with ADHD. The aim of this study is to compare the (cost) effectiveness of mindfulness training to the (cost) effectiveness of methylphenidate in children with ADHD on measures of attention and hyperactivity/impulsivity.

**Methods/design:**

A multicenter randomized controlled trial with 2 follow-up measurements will be used to measure the effects of mindfulness training versus the effects of methylphenidate. Participants will be youngsters (aged 9 to 18) of both sexes diagnosed with ADHD, referred to urban and rural mental healthcare centers. We aim to include 120 families. The mindfulness training, using the MYmind protocol, will be conducted in small groups, and consists of 8 weekly 1.5-h sessions. Youngsters learn to focus and enhance their attention, awareness, and self-control by doing mindfulness exercises. Parents will follow a parallel mindful parenting training in which they learn to be fully present in the here and now with their child in a non-judgmental way, to take care of themselves, and to respond rather than react to difficult behavior of their child. Short-acting methylphenidate will be administered individually and monitored by a child psychiatrist. Assessments will take place at pre-test, post-test, and at follow-up 1 and 2 (respectively 4 and 10 months after the start of treatment). Informants are parents, children, teachers, and researchers.

**Discussion:**

This study will inform mental health care professionals and health insurance companies about the clinical and cost effectiveness of mindfulness training for children and adolescents with ADHD and their parents compared to the effectiveness of methylphenidate. Limitations and several types of bias that are anticipated for this study are discussed.

**Trial registration:**

Dutch Trial Register: NTR4206. Registered 11 October 2013.

## Background

Attention-Deficit-Hyperactivity-Disorder (ADHD) is one of the most common childhood disorders, with a prevalence of 5 % [1]. Children and adolescents with ADHD show inattentive, impulsive, and hyperactive behavior that interferes with their (social) functioning or development [1] and occurs in more than one setting (e.g. in social situations, at school, at work, or at home). Following the diagnostic criteria of the fifth edition of the Diagnostic and Statistical Manual of Mental Disorders (DSM-5) [1] inattentive behavior refers to difficulties with organizing and planning tasks or activities and with maintaining attention over prolonged periods of time, such as wandering off during tasks or lacking persistence. Examples of hyperactive behavior are running and climbing in inappropriate situations, fidgeting or tapping with hands or feet, and excessive talking. Impulsivity refers to difficulties with inhibiting proponent responses, such as interrupting or intruding on others’ conversations or activities, answering before a question has been completed, and making important decisions without forethought. Depending on which key symptom is most present, three types of ADHD classifications can be distinguished: a predominantly inattentive presentation (also known as Attention Deficit Disorder, ADD), a predominantly hyperactive/impulsive presentation, or a combined presentation.

It has been demonstrated that children and adolescents diagnosed with ADHD have a substantial economical impact on society [2–5]. A meta-analysis [2] reviewed seven European-based studies and found that the average total annual costs related to childhood ADHD lie between €9,860 and €14,483 per patient, and national annual costs ranged from €1,041 to €1,529 million. With 648 million, most costs were related to education. Health care costs for childhood ADHD were estimated between €87 and €377 million, and social services costs were €4.3 million per year. From a family perspective, family members of children and adolescents with ADHD add to the economical burden with €161 million of health care costs, and with €143 to €339 million because of productivity losses.

Medication and psychosocial interventions are the most commonly used treatments for reducing ADHD symptoms in children and adolescents [6]. Regarding medication for ADHD, psychostimulants, especially methylphenidate, is globally the most prescribed drug [7] and is being used increasingly since the 1990′s, with a calculated global consumption of 72 tons (2.4 billion defined daily doses for statistical purposes) of methylphenidate in 2013 [8]. Over the years, the highest consumption of methylphenidate took place in the United States. However, since 2000 many other countries, including the Netherlands, show a sharp increase in the use of methylphenidate as well [9, 10]. In the Netherlands 130,000 youngsters were using methylphenidate in 2012 [11], which was at the time 3.2 % of Dutch youngsters [12]. In 2014 the largest group of methylphenidate users were children with ADHD between 11 and 14 years [13], more than 70 out of 1000 children in this age category with ADHD were using methylphenidate. Although the amount of diagnoses in the Netherlands increased over the years, and therefore the use of medication as well, the percentage of children on medication remains stable, which is about two-thirds of the children diagnosed with ADHD and one-third of the children diagnosed with ADD [12]. Many studies have shown that methylphenidate is effective in the treatment of childhood ADHD [14–16] and that, when controlled for placebo effects, it has beneficial effects for about 70 % of the children with ADHD [17–19]. According to international guidelines it is recommended to prescribe methylphenidate as a first drug of choice when pharmacological treatment is indicated [20, 21]. Only when this drug does not reach its intended effects, guidelines advise to move on prescribing other medication (mainly dextroamphetamine and atomoxetine). International guidelines further advise that pharmacological treatment should always be part of a more comprehensive treatment program that includes psycho-education and may include behavioral treatment, parent training, and/or teacher-administered behavior therapy [21–23]. However, guidelines of the American Academy of Child and Adolescent Psychiatry (AACAP) [22] also suggest that when a patient with ADHD experiences robust beneficial effects from pharmacological treatment, and therefore shows normal functioning in several life domains, that this treatment alone is satisfactory. This recommendation is supported by randomized controlled trials (RCTs) such as the Multimodal Treatment of ADHD (MTA) study [24] and a meta-analysis [6], comparing methylphenidate with psychosocial treatment and their combination.

In MTA study [24] 579 children were randomized over 14 months of methylphenidate treatment, intensive behavioral treatment, a combination of these two treatments, or standard community care. Children receiving combined treatment and medical treatment showed a larger decline in ADHD symptoms compared to children receiving behavioral treatment or community care. Moreover, the combined treatment did not have an additive effect in reducing ADHD symptoms compared to medical treatment alone. Van der Oord et al. [6] compared 24 studies, including the MTA study, about the effectiveness of methylphenidate, psychosocial treatment, or their combination in children with ADHD. It was concluded that both methylphenidate and psychosocial treatment were effective in reducing ADHD symptoms, but that psychosocial treatment alone had smaller effects than methylphenidate and a combined treatment. Similar to the findings of the MTA study, in this meta-analysis psychosocial treatment did not show to have additive value to methylphenidate in recuding ADHD sympotms either. Another meta-analysis [25] compared randomized controlled studies evaluating the effects of non-pharmacological treatment for ADHD, both dietary interventions (Restricted Elimination Diets; *n* = 7, artificial food color exclusions; *n* = 8, and free fatty acid supplementation; *n* = 11) and psychosocial interventions (cognitive training; *n* = 6, neurofeedback; *n* = 8, and behavioral interventions; *n* = 15). For all 6 types of interventions results illustrated a reduction in core ADHD symptoms when rated by a person (often unblinded) closest to the therapeutic setting. However, when ratings from persons blind to the treatment condition were evaluated, only free fatty acid supplementation and artificial food color exclusion remained effective in reducing core ADHD symptoms. The authors concluded that the effect sizes found for non-pharmacological treatments are substantially lower than those found in studies on ADHD medication and that better evidence from blinded assessments is needed for psychosocial interventions for ADHD in order to be offered as evidence-based treatments. In an earlier meta-analysis [26], 174 studies on the effectiveness of psychosocial interventions (parent-based, teacher-based, and child-based) for children with ADHD were included. The overall results show that psychosocial interventions are effective in reducing ADHD symptoms and that effect sizes found in this study are comparable to those found for stimulant medication for ADHD. The difference between the latter two meta-analyses is, however, that Sonuga-Barke et al. [25] only included RCTs falling into the highest category of evidence, that is evidence from at least one RCT [27], whereas Fabiano et al. [26] also included studies falling into lower categories of evidence (e.g. uncontrolled studies and single-case studies). Besides, Fabiano et al. [26] included children with externalizing behavior problems but without a diagnosis of ADHD, which may explain part of the highly positive outcomes as well. Lastly, a large recent review on the effects of methylphenidate alone for children and adolescents (*n* = 12.245, ages ranged from 3 to 21 years) with ADHD included 185 RCTs comparing methylphenidate versus placebo or no intervention [28]. Results show that methylphenidate may reduce the key symptoms of ADHD and may improve general behavior and quality of life. However, due to mostly poor designed research trials and, therefore, high risk of bias for all included studies, the quality of de evidence is low. Better designed RCTs, especially regarding the blinding process, are needed to further establish the evidence of the effectiveness of methylphenidate. Moreover, the authors stress the importance for large RCTs of non-pharmacological treatments for ADHD.

In sum, the international guidelines for treatment of ADHD, supported by the current knowledge about the effectiveness of methylphenidate compared to the somewhat more ambivalent evidence of the effectiveness of other treatment options, suggest that methylphenidate for children with ADHD is, to date, still the first-line treatment [29]. Moreover, looking at the cost effectiveness of medication versus behavioral treatment, medication also seems to be the preferred option as it was estimated that medical costs per child with ADHD is $1079 during a period of 14 months, whereas costs for behavioral treatment per child with ADHD is $7176 during that same period of time [30]. Nevertheless, concerns about the frequency of methylphenidate prescriptions and its possible disadvantages are rising increasingly [8, 31]. These concerns are with good reason, given the literature on the substantial limitations of (stimulant) medication for ADHD. First, usage of stimulant medication may result in side effects such as insomnia, loss of appetite, abdominal pain, headache, anxiety, stress, and nervousness [14, 20, 28, 31, 32]. In the MTA study [24] 64.1 % of the children suffered from one or more mild, moderate, or severe side effects. Second, stimulant medication works only short-term and symptoms return once medication is stopped [20, 33, 34]. Therefore, children with ADHD must continue the use of medication for extended periods of time in order to maintain the beneficial effects [35]. Third, as previously stated, about 70 % of children with ADHD show a symptomatic response to methylphenidate, however, up to 30 % of the children do not benefit from methylphenidate at all [17, 18, 36, 37]. When other pharmacological treatments for ADHD are systematically administered, still 10 % of the children do not respond to any of the medications [24]. Fourth, treatment fidelity is often low with nonadherence rates between 13.2 to 64 % in people with ADHD [38]. Nonadherence is greater for short-acting stimulants compared to long-acting stimulants [7]. Nonadherence may be due to inadequate supervision including delayed or missed doses, but also because patients may forget or refuse to take medication [7]. The most prescribed stimulants are short-acting, including methylphenidate, and require intake of 2 or 3 times a day. As a consequence children need to take medication in public, for example at school, which may be embarrassing or (socially) stigmatizing [7, 39]. Fifth, stimulant medication is a contraindication for people with schizophrenia, hyperthyroidism, cardiac arrhythmias, angina pectoris, and glaucoma. Furthermore, extra caution needs to be taken in case of hypertension, depression, tics, epilepsy, anorexia, autism spectrum disorders, severe mental retardation, or a history of drug abuse or alcoholism [20]. Sixth, the safety of medication for children with ADHD is not fully known [31, 40]. Whereas short-term side effects may be reversible when medication is stopped, little is known about long-term side effects. There is limited literature of the impact of long-term medication use on growth, blood pressure, heart rate, and the occurrence of suicidal, psychotic, and manic symptoms [40]. Some studies found that children with ADHD who take medication for several years show reduced growth and weight compared to their peers [41]. The difference in growth, however, seems to attenuate over time and there is debate about whether the ultimate adult growth is affected. Seventh, the effectiveness of long-term use of methylphenidate is not fully known [40]. Studies on the effectiveness of ADHD medication show robust effects on symptom reduction and other life functioning domains up to 2 years later [42]. So far, little is known about the effectiveness beyond this period. However, results of the MTA study 8-year follow-up data failed to demonstrate the benefits of medication treatment beyond 2 years for most of the children [43].

Because of the above named limitations and uncertainties, children and their parents may not view medication as a considerable option. They are not open to try medication but would like to receive non-pharmacological treatment [7]. To conclude, medication is worldwide the primary treatment of choice for children with ADHD, but has enormous disadvantages, and psychosocial treatments, so far, failed to demonstrate sufficient efficacy. Therefore, there is a large demand for alternative treatment options. Mindfulness training became increasingly popular in the last decade, with studies showing promising results in this burgeoning field, and is for many reasons a potential contender in the treatment for childhood ADHD.

Mindfulness training is an intervention based on Eastern meditation techniques, that aims to increase awareness by paying attention on purpose in the present moment, enhance non-judgmental observation, and reduce automatic responding [44]. Individuals are encouraged to direct their attention towards internal experiences such as bodily sensations, emotions, thoughts, and action tendencies, as well as to environmental stimuli such as smells and sounds in their surroundings [45]. The ability to focus and sustain attention in the present moment and to bring back the attention to the present moment whenever it wandered off, which is trained during a mindfulness course, may be especially beneficial for children diagnosed with ADHD, as 1 of the core symptoms of ADHD is inattention. Practicing mindfulness may give children more control over their attention, which may, in turn, be beneficial for other psychological symptoms as well [46–48]. Furthermore, the ongoing streams of internal and external stimuli that enter 1’s awareness are to be observed without evaluating or judging them [45]. By doing so, 1 learns -by first person experience- to be accepting of whatever is present, independent from the valence of the stimulus. Patterns of thoughts, emotions, and reactions will be recognized, and hence, by consciously bringing attention to them, these automatic patterns can be interrupted. Individuals learn to respond rather than to react to stimuli. This ability also may be especially beneficial for children diagnosed with ADHD, as the other core symptom is hyperactive and impulsive behavior. By noticing which impulses are arising or the tendency to react hyperactive, 1 creates the possibility to choose how to respond, rather than to react on automatic pilot.

Mindfulness meditation has been incorporated into programs such as Mindfulness Based Stress Reduction (MBSR) [49] and Mindfulness Based Cognitive Therapy (MBCT) [50]. MBSR was originally developed for chronic pain patients in order to help them cope with their illness, whereas MBCT (mindfulness meditation incorporated with cognitive therapy) was developed as a relapse prevention method for patients suffering from recurring depression. Evidence from a large number of studies suggests that mindfulness based interventions are associated with positive psychological effects, such as improved well-being, quality of life, and regulation of behavior, and reduced psychopathology and emotional reactivity [47]. Strong evidence for the effectiveness of mindfulness in reducing depression, anxiety, and stress in adults exists [50–53]. Moreover, preliminary evidence from mindfulness studies suggests a reduction in physical complaints, such as (chronic) pain and somatization disorders [51, 54–56]. Gu, Strauss, Bond, and Cavanagh [57] conducted a meta-analytic review about which mechanisms of change underlie improved mental health and wellbeing in adults who followed a mindfulness based intervention. Results evidence that the effects of mindfulness based interventions indirectly improved mental health (e.g. depression, stress, anxiety, mood states, and negative affect) through changes in cognitive and emotional reactivity, mindfulness, and repetitive negative thinking. Preliminary but insufficient evidence was found for self-compassion and psychological flexibility as mechanisms of change. However, another study did find evidence that self-compassion is a mediating mechanism in MBCT’s treatment outcomes [58].

Although the effects of mindfulness training in adults are well established, research on the effectiveness of mindfulness training in child and adolescent psychiatry is a relatively new domain. The majority of research in this field addresses children and adolescents in non-clinical samples [46]. The meta-analysis conducted by Zoogman et al. [46] included 20 studies on mindfulness based interventions with youth, of which four were clinical studies. Results show a small to moderate universal effect size for all mindfulness interventions taken together (*del* = 0.23), surpassing the effects of active control groups. Moreover, findings suggest that mindfulness training may be more beneficial for clinical samples than for non-clinical samples, and also more effective in reducing symptoms of psychopathology than other outcome measures. These studies show preliminary evidence that mindfulness based interventions are also beneficial for youth with a variety of psychological symptoms, as improvements were reported on measures of attention, internalizing and externalizing behavior problems, sleep, anxiety, and academic performance.

Regarding studies that specifically focused on the effects of mindfulness training for children and adolescents with ADHD (and their parents), so far, 8 studies have been performed.

The study of Bögels et al. [59] included 14 clinically referred adolescents (aged 11 to 18) suffering from externalizing disorders and their parents, of which two adolescents had a primary ADHD diagnosis and another two had co-morbid ADHD. The adolescents followed an early version of the 8-week MYmind mindfulness training with a parallel mindful parenting training for their parents (Bögels SM. MYmind: a mindfulness training for children with ADHD and their parents. In preperation). Adolescents and their parents were measured at waitlist, pre-test, post-test, and at 8-week follow-up. After the training, adolescents reported a substantial improvement on personal goals, internalizing, externalizing, and attention problems, happiness, and mindful awareness, and scored substantially higher on the d2 Test of Attention. In turn parents reported at post-test an improvement in their adolescents goals, externalizing and attention problems, self-control, attunement to others, and withdrawal. These effects were maintained at 8-week follow-up.

In the study of Singh et al. [60], two children with ADHD (aged 10 and 12) and their mothers participated. Children received a 12-session mindfulness training parallel to the mindful parenting training of their mothers, using a multiple baseline across mothers and children design. Mothers reported an improvement in compliance by their child as a result of the mindful parenting training, compliance was further increased by the child training. Results were maintained during the 24-week follow-up. Moreover, their results evidenced an improved mother-child interaction and satisfaction with their parenting. Children in this study were only assessed on a behavioral outcome, but not on core symptoms of ADHD.

Zylowska et al. [61] conducted a feasibility study with a pre- and post-test design, with 24 adults and 8 adolescents with ADHD, who followed an 8-week mindfulness training adapted for ADHD. After the training participants reported a decline in self-reported ADHD symptoms, but not hyperactivity, and improvements on neurocognitive tasks for measures of attentional conflict, but not working memory. In adults improvements were found in anxiety and depression. Due to the low numbers in this study no separate conclusions were drawn for adolescents alone.

In a study of Haydicky et al. [62] effects of a 20-week Mindfulness Martial Arts training were evaluated in 60 children in a clinical sample of adolescent boys (aged 12-18) with learning disabilities, using a pre-and post-test design and a waitlist control group. Twenty-eight participants were diagnosed with co-occurring ADHD of which 14 were assigned to the mindfulness training and 14 to the waitlist control group. Findings in this subgroup showed a decrease in parent-rated externalizing behavior, oppositional defiant problems, and conduct problems. In another study of Haydicky et al. [63] effects of the 8-week MYmind mindfulness training, for adolescents with ADHD (*n* = 18, aged 13-18) and a parallel mindful parenting training for their parents (*n* = 17), were evaluated using a pre-post-follow-up design and a within-group waitlist control without randomization. At post-test adolescents did not report improvements on any of the measures. However, parents reported a decline in their adolescents’ inattention, conduct problems, and peer relationship problems and in their own parenting stress. Parents also reported an increase in their mindful parenting. Generally, gains achieved during the training were maintained at the 6-week follow-up and adolescents now reported a decrease in their own internalizing problems.

Another study measured the effects of the MYmind mindfulness training for 13-18 year old adolescents with ADHD (*n* = 9) and a parallel mindful parenting training for their parents (*n* = 13), using a time-series design during baseline, the training, and six months follow-up [64]. Results showed a decline in parent and adolescent stress, and parent and adolescent distress due to family conflict. Parents, but not adolescents, reported a reduction in adolescents’ inattention, hyperactivity, and impulsivity. These improvements were generally maintained at follow-up six months later.

Finally, two studies were conducted by Bögels and colleagues. The first study [65] assessed the effects of an early version of the 8-week MYmind mindfulness training for children with ADHD (*n* = 22, aged 8-12) with parallel mindful parenting training, using a pre-post-follow-up design and a within-group waitlist control without randomization [65]. Results showed a significant reduction of parent-rated ADHD behavior of themselves and their child, which was maintained at follow-up. Furthermore, a significant reduction of parental stress and over-reactivity at follow-up was shown. The second study assessed the effects of an early version of the MYmind mindfulness training for adolescents with ADHD (*n* = 10, aged 11–15) with parallel mindful parenting training, using a pre-post-follow-up design without randomization [66]. Findings showed a reduction of self-reported ADHD behavior in adolescents and improvements on objective neuropsychological computerized tasks of attention. Based on fathers’ and teachers’ reports a decline in ADHD behavior in adolescents was shown. Fathers reported reduced parenting stress as a result of the mindful parenting training and mothers reported a decline in parental over-reactivity. At 8-week follow-up the effects were even stronger than at the post-test, however, the effects waned off at 16-weeks follow-up.

In sum, preliminary effectiveness of mindfulness training for children and adolescents with ADHD is clearly demonstrated in the above-mentioned studies. However, the current stage of research in this field is limited by a lack of randomized and controlled (clinical) trials with large samples, standardized formats for interventions, objective measures, and that are generalizable outside the intervention context [46, 67]. Therefore, it is a logical step to further assess the (cost-) effectiveness of mindfulness training, in children and adolescents with ADHD, in a well-designed RCT with a large number of participants, in which mindfulness training is evaluated against methylphenidate, the current treatment of choice for childhood ADHD.

### Objectives

The primary objective of this RCT is to compare mindfulness training with the currently most effective treatment, methylphenidate, for children with ADHD. To the scope of our knowledge these two treatments have never been compared before in an RCT concerning children with ADHD. Effects of mindfulness training for children combined with mindful parenting training on the primary outcome measures of attention and hyperactivity/impulsivity are compared to those of methylphenidate in children and adolescents with ADHD. In addition, we will compare the effectiveness of mindfulness training versus methylphenidate with respect to: 1) cost-effectiveness; 2) secondary child measures: a) psychopathology, b) stress, c) quality of life, d) happiness, and e) sleep (problems); 3) secondary parent measures: a) parents’ own ADHD and psychopathology, b) stress, c) quality of life, d) sleep (problems), and e) parenting sense of competence; and 4) potential mechanisms of change: a) mindful awareness (of parents and children in general, of parents in their parenting role, and parental self-compassion), b) emotion regulation (child self- and emotion regulation, and family emotion regulation), and c) parenting (parenting style and mind mindedness). Additionally, treatment adherence (attendance to weekly sessions by parent and child and minutes of home practice by parent and child) will be monitored.

## Methods/design

### Trial design

**Fig. 1:**
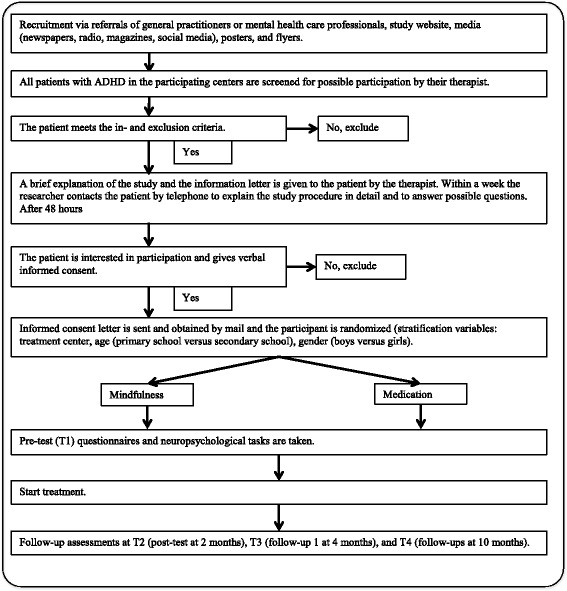
Flow Chart of recruitment and study procedure

A multicenter RCT with follow-up measurements is used to measure the effects of mindfulness training versus the effects of methylphenidate. After enrolment in the study participants sign informed consent and are randomized to the mindfulness arm or the methylphenidate arm. After randomization, participants fill in the pre-test (T1) and start the treatment they were assigned to. In the mindfulness arm participants receive 8 sessions of mindfulness training (1 session per week) and in the methylphenidate arm participants start the first 8 weeks of methylphenidate intervention. After those 8 weeks, participants fill in the post-test (T2). Subsequently, for participants in the mindfulness arm, 8 weeks without training follow after T2, and receive a booster session at the end of those 8 weeks. Participants in the methylphenidate arm continue taking methylphenidate for another 8 weeks. Four months (T3) and ten months (T4) after participants started their treatment, follow-up measurements are planned in order to determine whether training effects are long lasting. Between T3 and T4, a 6 month-period passes in which participants do not have to be in treatment, but they are free to consider other treatment options. Thus, participants in the methylphenidate arm can decide to continue their medication, change medication, stop medication, or to remain without treatment, or get enrolled in mindfulness training or another intervention. Participants in the mindfulness arm can decide to remain without treatment, start medication or participate in another intervention. See Fig. 1 for a flow chart of recruitment and study procedures.

### Preference study

It is anticipated that, given the differences in nature of both treatments, a proportion of potential participants have a strong treatment preference and are, therefore, not willing to be randomized. For this reason a parallel preference study, in which participants choose for one of the two treatments, is conducted in addition to the RCT. This study is approved by the Ethics Review Board of the Faculty of Social and Behavioral Sciences at the University of Amsterdam (no. 2014-CDE-3658). The preference study has similar objectives as the RCT and participants follow, except for randomization, the exact same procedures as participants in the RCT. However, due to expected preference bias in the preference study data is collected and analyzed separately.

### Participants

Participants (*n* = 120) are children and adolescents between 9 and 18 years of age of both sexes diagnosed with ADHD. When children are 8 years old but already in the fifth year of primary school, they are also allowed to participate, since we assume that they are mentally ready to understand the instructions of the mindfulness training. Participants are recruited via referrals of general practitioners or mental health care professionals, study website, media (newspapers, radio, magazines, social media), posters, and flyers. Participating in this study is completely voluntary and participants are free to withdraw from the study and/or the treatment at any moment without having to give a reason and without any consequences for further treatment.

Inclusion criteria are (1) the child has a primary DSM classification of ADHD, (2) ADHD is verified by the child/adolescent version of the Structured Clinical Interview for DSM 5 (SCID-Junior) [68], (3) the child is between 9 and 18 years of age, (4) (estimated) IQ is over 80, and (5) at least one parent is willing to participate in the mindful parenting training and to accompany their child to the medical consultations. Participants are excluded from participation in case of (1) inadequate mastery of the Dutch language by the child or parents, (2) suffering from psychosis, schizophrenia, or untreated Post Traumatic Stress Disorder (PTSD), (3) comorbid conduct/behavior problems that are so severe, already during intake, that interaction/talking between parent and child is not possible, (4) current or previous use of methylphenidate in the past year, (5) current or previous participation in mindfulness training in the past year, and (6), participation in a currently active other psychological intervention. Only the first exclusion criterion applies to both parents and children, the last 5 apply to the children solely.

To verify the inclusion criterion ‘(estimated) IQ is over 80’, an abbreviated version of the Dutch version of the Wechsler Intelligence Scale for Children – Third edition (WISC-III-NL) [69, 70] is used. Two subtests (Vocabulary and Block Design) were selected [71]. This task is performed during T1 after the neuropsychological attention tasks are completed.

### Assessment of eligibility and consent to participate

Psychologists at the two participating treatment centers assess every patient with ADHD for eligibility following the in- and exclusion criteria of this study. When eligible, patients are given the information letter about the content of the study. About a week later, eligible patients are contacted by phone by the researcher, the study procedures are explained, and families are asked for study participation. When verbal consent is obtained, participants are sent the written informed consent on paper, which they can return by mail or hand over during the first appointment with the researcher. Parents sign the informed consent for their own and their child’s or adolescent’s participation. Adolescents (12+) are also requested to sign their own informed consent.

### Study settings

The study will be conducted at the urban treatment center UvA minds and the more rural treatment center Buro van Roosmalen. Both centers are outpatient academic treatment centers in the Netherlands, working with children and adolescents with psychiatric problems, and/or family problems.

### Interventions

Before children and their parents start the treatment to which they are assigned to, they receive individual psycho-education of 2 to 3 sessions by their psychologist of the treatment center.

### MYmind training

The mindfulness training is conducted in groups of 4 to 6 participants (children) or 6 to 8 participants (adolescents), and consists of 8 weekly 1.5-h sessions. Eight weeks after the training both children and their parents receive a 1.5-h follow-up session. For the training the MYmind protocol is used: Mindfulness training for Youngsters with ADHD and their parents [59, 65, 66]. Participants learn to focus and enhance their attention, awareness and self-control by doing mindfulness exercises during the training and home practice. Both parents and children are asked to practice daily meditations. Parents follow parallel mindful parenting training (in a group, 1.5 h/week) based on the protocol described by Bögels and Restifo [72]. Parents learn to be fully present in the here and now with their child in a non-judgmental way, to take care of themselves, and to respond rather than react to difficult behavior of their child. They also learn how to guide their child in the meditation. The importance for parents to practice daily, in order for them to embody mindfulness, to function as a role model, as well as for their own inattention and impulsivity/hyperactivity symptoms, is emphasized. Although most of the training time children and parents are in separate groups, the first half hour of session 1 and 5, and most of session 8 and the follow-up session take place with parents and children together, in order to share experiences. Themes addressed in the MYmind training for children with ADHD and their parents are: 1) Beginners’ mind, 2) Home in our body, 3) The breath, 4) Distractors!, 5) Stress, 6) High way, walking way, 7) Acceptance & autonomy, and 8) The future, and for the follow-up session: Each time, beginning a new (Bögels SM. MYmind: a mindfulness training for children with ADHD and their parents. In preperation). Participants are free to discontinue attending the mindfulness sessions on request.

Adherence to the homework practices of the mindfulness training is assessed during the eight weeks of the training and the next eight weeks of self-practice using a registration form on which both parents and children fill out their practices on a daily basis. Sessions are videotaped and will be scored on integrity. The MYmind training is delivered by experienced mindfulness trainers who are trained in the MYmind program by the third author, who also provides regular supervision.

### Methylphenidate

Short-acting methylphenidate is administered individually and monitored by a child psychiatrist, following the guidelines of “Multidisciplinary guidelines ADHD” [73]. After the first face-to-face consultation with the child psychiatrist, in which children are physically and psychologically examined, they receive a prescription of 2.5 or 5 mg of short acting methylphenidate. After one week the psychiatrist contacts the child or his/her parent (s) by phone, when the medication already has the desired effect, the dose is not increased. However, when the medication is not yet effective, the dose is increased by 2.5 or 5 mg. Every time the dose is increased, the psychiatrist contacts the child or his/her parent (s) by phone one week later to evaluate the effects and, if needed increases the dose with another 2.5 or 5 mg with a maximum of 20 mg methylphenidate per dose until optimal titration is achieved. Every four weeks the child and at least one of his/her parents are invited to come to the treatment center for a face-to-face consultation with the child psychiatrist and a physical examination. All children start with three doses of 2.5 or 5 mg methylphenidate per day, morning, early afternoon, and late afternoon, seven days a week. In this manner both parents and teachers at school have the chance to observe changes in the child. After two or three weeks, in consultation with the child psychiatrist, children and their parents can chose to limit the dose frequency to two times a day and/or on weekdays only. Participants are free to discontinue methylphenidate use on request. When methylphenidate is not effective or severe side effects emerge, change in dose or medication type will be made after consultation with the psychiatrist or medication use will be discontinued completely.

Adherence to the methylphenidate treatment is assessed throughout the 16 weeks of the treatment using a registration form filled in on a daily basis by the parent and/or the child.

### Outcome measures

Tables 1 and 2 display an overview of the measures and measurement occasions in this study. Participants (i.e. children, mothers, fathers, and teachers) receive links to the online questionnaires, researchers fill out a questionnaire on paper. Neuropsychological attention tasks are administered at the treatment center, after which the child receives a 5-euro voucher for the effort.

**Table 1:** Child measures

Measure	Target concept	T1	T2	T3	T4
Children’s report					
YSR ^a^	Children’s psychopathology	x	x	x	x
SVK	Perceived stress	x	x	x	x
FEEL-KJ	Emotion regulation	x	x	x	x
HSR ^a^	Healthy self-regulation	x	x	x	x
SHS	Happiness	x	x	x	x
CAMM ^a^	Acceptance and Mindfulness	x	x	x	x
WHO5	Quality of life	x	x	x	x
CSRQ	Sleeping reduction	x	x	x	x
Sleep efficiency	Sleep efficiency	x	x	x	x
Sleep quality	Sleep quality	x	x	x	x
HSDQ	Restless leg syndrome (subscale)	x	x	x	x
EQ-5D-3 L	Quality of life	x	x	x	x
(Neuro) psychological tests					
TEA-Ch	Attention	x	x		
D2 test of attention	Attention	x	x		
Emotion Discussion Task	Emotion regulation	x	x		

*Note*: ^a^ Only filled out by children who are 11 years and above. T1 = pre-test, T2 = post-test, T3 = follow-up 1, T4 = follow-up 2

**Table 2:** Parent, teacher, and researcher measures

Measure	Target concept	T1	T2	T3	T4
Parents’ report					
DBDRS	Behavioral problems (ADHD)	x	x	x	x
CBCL	Children’s psychopathology	x	x	x	x
ASR^a^	Parents’ psychopathology	x	x	x	x
ZVAH	Self-reported ADHD	x	x	x	x
PSS^a^	Perception of stress	x	x	x	x
PSOC^a^	Parenting Sense of Competence	x	x	x	x
PS^a^	Parenting styles	x	x	x	x
FFMQ^a^	Facets of mindfulness	x	x	x	x
IM-P^a^	Mindful parenting	x	x	x	x
SCS^a^	Self-compassion	x	x	x	x
FER^a^	Family emotion regulation	x	x	x	x
MM	Mind Mindedness	x	x	x	x
WHO5^a^	Quality of life	x	x	x	x
PSQI	Sleep quality	x	x	x	x
HSDQ^a^	Insomnia (subscale)	x	x	x	x
EQ-5D-3 L	Quality of life	x	x	x	x
Cost-Questionnaire	Cost effectiveness	x	x	x	x
Emotion Discussion Task	Emotion regulation	x	x		
Teacher’s report					
TRF	Children’s psychopathology	x	x		
DBDRS	Behavioral problems (ADHD)	x	x		
Researcher’s report					
TOF	Children’s psychopathology	x	x		

*Note*: ^a^ Only filled out by the parent who is participating in the parallel mindful parenting training or who is accompanying their child at the psychiatrist. T1 = pre-test, T2 = post-test, T3 = follow-up 1, T4 = follow-up 2

### Primary outcome measures

The primary outcome of this study is ADHD symptoms (inattention and hyperactivity/impulsivity) as reported by multiple informants and measured on neuropsychological tasks. ADHD symptoms are measured on 2 subscales (Inattention Symptoms and Hyperactivity/impulsivity Symptoms) of the Dutch parent/teacher version of the Disruptive Behavior Disorder Rating Scale [74] (DBDRS, 42 items on a 4-point scale), and on the subscale attention problems of the Dutch versions of the Child Behavior Checklist [75] (CBCL, 113 items on a 3-point scale), the Teacher’s Report Form [75] (TRF, 113 items on a 3-point scale), the Youth Self Report [75] (YSR, 112 items on a 3-point scale), and the Test Observation Form [76] (TOF, 125 items on a 3-point scale). Furthermore, neuropsychological estimation of selective attention, sustained attention, directed attention, and attentional control are measured on 4 subtasks (Sky Search, Score!, Creature Counting, and Same Worlds) of the Test of Everyday Attention for Children [77, 78] (TEA-Ch) and on the D2 Test of Attention [79].

### Secondary outcome measures for children

Psychopathology is measured on the broadband scales (internalizing and externalizing problems) of the CBCL [75], TRF [75], YSR [75] and TOF [76]. Behavior problems are measured on the subscales Conduct Disorder and Oppositional Defiant Disorder of the DBDRS. Stress is measured on the Stress Questionnaire for Children [80] (SQ-C, 19 items on a 4-point scale). Quality of life is measured on the World Health Organization Well-Being Index [81] (WHO-5, 5 items on a 5-point scale). Happiness is measured on the Subjective Happiness Scale [82] (SHS, 4 items on a 7-point scale). Chronic sleep reduction is measured on the Chronic Sleep Reduction Questionnaire [83] (CSRQ, 20 items on a 3-point scale). Sleep efficiency and sleep quality are measured on a 15-item (8 open ended questions and 7 questions on 3-point scale) self-report questionnaire, as described in Meijer and van den Wittenboer [84]. Restless leg syndrome is measured on the Restless Leg Syndrome subscale of the Holland Sleep Disorders Questionnaire [85] (HDSQ, 5-item subscale on a 5-point scale).

### Secondary outcome measures for parents

ADHD symptoms of parents are measured with the Self-report Questionnaire for Inattention and Hyperactivity [86] (SQIH, 23 items on a 4-point scale). Other symptoms of parental psychopathology are examined using the broadband scales (internalizing and externalizing problems) of the Adult Self Report (ASR, 126 items on a 3-point scale). Stress is measured by the Perceived Stress Scale [87, 88] (PSS, 10 items on a 5-point scale). Quality of life is assessed with the WHO Well-Being Index [81] (WHO-5, 5 items on a 5-point scale). Sleep quality and efficiency are measured by the Pittsburgh Sleep Quality Index [89] (PSQI, 8 open ended questions and 14 items on a 4-point scale). Insomnia is assessed using the subscale Insomnia of the HDSQ [85] (8-item subscale on a 5-point scale). Parenting competence is examined with the Parenting Sense of Competence [90, 91] (PSOC, 17 items on a 6-point scale).

### Mechanisms of Change

Mindful awareness (of child and/or parent) as a possible working mechanism underlying the effect of the MYmind training and the mindful parenting training is measured by a) the Children’s Acceptance and Mindfulness Measure [92, 93] (CAMM, 10 items on a 5-point scale), b) the 5 Facet Mindfulness Questionnaire [94, 95] (FFMQ, 24 items on a 5-point scale), c) the Interpersonal Mindfulness in Parenting scale [96, 97] (IM-P, 31 items on a 5-point scale), and d) the Self Compassion Scale [98] (SCS, 12 items on a 7-point scale).

Further, child and family emotion regulation as a potential mechanism of change is measured by a) the Healthy Self-Regulation Subscale [99] (HSR, 12 items on a 6-point scale), b) the FEEL-KJ [100] (90 items on a 5-point scale), c) the Family Emotion Regulation scale (FER, 11 items on a 6-point scale), which is an adaptation of the HSR [99] for families, and d) the Emotion Discussion Task [101] (EDT, videotaped and coded on a 5-point scale), in which the parent and the child are asked to discuss a time (5 min each) when the child felt anxious, angry and happy.

The last hypothesized working mechanism, parenting, is assessed by a) the Parenting Scale [102, 103] (PS, 30 items on a 7-point scale) and b) Mind Mindedness [104] (MM) which is measured with a single item question in which the parent is asked to describe their child in a minimum of 10 sentences.

### Cost-effectiveness

The economic evaluation will be performed from a societal perspective, with a time horizon of 40 weeks and will follow the methodology applied in Van Steensel, Dirksen, and Bögels [105]. Incremental Cost-Effectiveness Ratios (ICER) will be expressed as: 1) Clinical significant improvement on ADHD symptoms in children, (2) The incremental costs per Quality Adjusted Life Years (QALY), and (3) ADHD symptom free families.

The cost questionnaire will be completed covering two months retrospectively by the participating parent (s), at T1, T2, T3, and T4. As there are six months between T3 and T4 and the cost questionnaire only covers two months, the cost questionnaire will also be completed twice in between these two measurement occasions. For the cost questionnaire a modified version, specially adapted to families with children with ADHD, of the cost questionnaire applied in Van Steensel et al. [105] will be used. For health-related quality of life, the EuroQol EQ-5D-3 L [106] will be administered at all measurement moments. The subclinical threshold of the subscale attention of the DBDRS, YSR, and SQIH will be used as variable to establish the presence of severe attention problems in both children and parents.

### Sample size

We expect methylphenidate to have a large effect on ADHD symptoms at T2 and T3 [6]. Based on pilot studies of our group [59, 65, 66] we expect effect sizes for mindfulness on ADHD symptoms to be medium to large at T2 and T3. With respect to current effect sizes found in the literature for mindfulness and methylphenidate, a medium to large difference in effect size is expected. To detect a difference of a medium effect between the mindfulness group and the methylphenidate group, a total sample size of 120 (60 in both arms) is needed with an anticipated power of 0.80, assuming a significance level of 0.05 for a 1-sided test of hypotheses and correlations of 0.5 between measurement occasions.

### Randomization

Participants are randomized over mindfulness (*n* = 60) and methylphenidate (*n* = 60). Stratified randomization is used to ensure that the ratio of boys and girls is equal in both the mindfulness group and the methylphenidate group. Randomization is carried out separately for UvA minds and Buro van Roosmalen and also separately for both age groups (children and adolescents). The first author will allocate participants to a treatment group using a computer-generated list of random numbers.

### Statistical analyses

Analyses will be conducted using the intention-to-treat principle. All participants who meet inclusion- and exclusion criteria, and who provided pre-training measurements will be included in the analyses. Also participants who refuse to complete measurements after T1 will be included in the analysis using multilevel modeling, which is robust against data that is missing at random [107]. Participants who drop out of treatment will be asked to complete all further measurements. Additionally, per-protocol analysis will be conducted. For the per-protocol analysis only the participants who adhered fully to the trial protocol will be included.

The primary research question can be answered through multilevel (mixed model) analysis, as data collected at different measurement occasions (T1, T2, T3, and T4) form a hierarchical structure of measurements nested within persons. Experimental effects are indicated by significant parameters for group by time interactions. Effects of mindfulness training on inattention and hyperactivity/impulsivity are compared to effects of methylphenidate on inattention and hyperactivity/impulsivity. Also effects of mindfulness training on the secondary outcome measures are compared to effects on methylphenidate using multilevel (mixed model) analysis. Impact of covariates, such as social economic status, expectations of how helpful the treatment will be, stratification factors (treatment center, age, and gender), and ADHD symptom severity at T1 will be evaluated. Effect sizes and corresponding 95 % confidence intervals will be presented. Background information (e.g. age, family situation, IQ/educational level, medication use or comorbid psychopathology) will be used for descriptive purposes.

### Mechanisms of Change

Possible underlying mechanisms of change, will be evaluated using a multiple mediation model following the principles and practice of structural equation modeling as described in Preacher and Hayes [108].

### Cost-effectiveness

The ICER and its 95 % confidence interval will be calculated using bootstrap analysis. This analysis results in a scatterplot of bootstrapped ICERs (i.e., cost-effectiveness plane) with four quadrants (1) bootstrapped ICERS falling in the north-east quadrant reflect higher effects costs for mindfulness compared to medication, (2) bootstrapped ICERs falling in the south-east quadrant reflect higher effects and lower costs for mindfulness compared to medication, (3) bootstrapped ICERs falling in the south-west quadrant reflect lower effects and costs for mindfulness versus medication, and (4) bootstrapped ICERs falling in the north-west quadrant reflect lower effects and higher costs for mindfulness compared to medication. In addition, acceptability curves will be obtained representing the chance that mindfulness is a cost-effective intervention compared to medication. Given the non-parametric approach of bootstrap analysis there is no required assumption about the distribution of the data [109]. The cost-analysis will be performed according to the Dutch guidelines for cost calculations [109] and includes all costs relevant to society.

### Handling and storage of data and documents

All participants will be assigned a unique code. Access to the key of this coding system (an SPSS coding file) is limited to the coordinating investigators and the principal investigator. When necessary, the auditor, the Medical Ethical Committee of the Amsterdam Medical Center, and employees of the Health Care Inspection (Inspectie voor de Gezondheidszorg) can access this coding system. On the informed consent papers, the name of the participant and the code of the participant are presented. Hence, the consent papers of the participants will be stored in a separate organizer, with access limited to the above mentioned persons. The questionnaires will be completed at home, after the participant is sent a link to the online questionnaire. This link is connected to the unique code of the participant. This way all the questionnaire data can be coded and analyzed anonymously.

### Compensation for injury

The study and the participating centres have a liability insurance which is in accordance with article 7, subsection 6 of the Medical Research Involving Human Subjects Act (WMO). The study also has an insurance which is in accordance with the legal requirements in the Netherlands (Article 7 WMO and the Measure regarding Compulsory Insurance for Clinical Research in Humans of 23th June 2003). This insurance provides cover for damage to research subjects through injury or death caused by the study. The insurance applies to the damage that becomes apparent during the study or within four years after the end of the study.

## Discussion

ADHD is a highly prevalent childhood disorder [1] and is associated with functional impairment [110] and a substantial economic impact [2]. Stimulant medication is effective in reducing ADHD symptoms [14–16] and is currently the first choice of treatment worldwide [29], but has limitations [31]. These limitations concern the severe side effects, low treatment adherence, short term effects, non-effectiveness, contraindications, and that its long-term effectiveness and safety are not yet well known. Psychosocial treatments show to be far less effective in reducing ADHD symptoms compared to stimulant medication and a combined psychosocial and medical treatment failed to show additive benefits to medication alone [6]. Therefore, there is a need for an alternative non-pharmacological treatment. The bourgeon of studies in the field of mindfulness treatment for childhood ADHD show promising results in reducing ADHD symptoms and may be a potential alternative to medication given that the nature of this treatment targets the core symptoms of ADHD. With this study we aim to compare the (cost) effectiveness of mindfulness treatment for children with ADHD and their parents to the effectiveness of treatment with methylphenidate in a RCT. Study outcomes, e.g. presented at conferences and published in scientific and peer reviewed journals, will inform children with ADHD and their families, general practitioners, and the mental health care sector about the effectiveness of mindfulness training and whether mindfulness is a considerable alternative to medication in the treatment of childhood ADHD. To the scope of our knowledge, a study about the cost effectiveness of mindfulness training for children with ADHD and their parents has not been conducted so far. Outcomes will also inform health insurance companies which treatment is more cost effective (in the long term). However, we expect, based on the cost-effectiveness analyses in the study of Jensen et al. [30] and due to the intensity of MYmind training and the relatively low costs of the medical treatment, that MYmind training will be less cost-effective than medication, even when medication is continued during the whole 40 weeks of the study.

An anticipated limitation of this study is a nonparticipation bias [111], as it is expected that a proportion of eligible participants has such a strong treatment preference that they are not willing to be randomized and, therefore, do not participate in this study. Those who are only open to medication or mindfulness treatment may differ in several characteristics from those who are willing to try both treatments. It is also anticipated that loss to follow-up bias will emerge during the course of the study for several reasons [112]. Participants who are most in need for treatment because of the severity of the symptoms may also be those who drop out first because the intensiveness of participating in the study or the assigned treatment becomes too much of a burden. Another example is that participants agreed to be randomized, but are unsatisfied with the assigned treatment and are, therefore, no longer willing to participate, which may lead to systematic missing values in one or both conditions. An example may be that patients suffering from severe ADHD symptoms will refuse a nonmedical treatment and that less severe cases may refuse medical treatment. Both forms of dropping out are violating the ecological validity. Sampling bias is another anticipated threat to the study as participants are excluded when they have used methylphenidate in the past year. Since many children with ADHD already are on medication, a large part of our population is excluded from participation. Children who meet the in- and exclusion criteria are probably mainly those who are recently diagnosed or those who’s symptoms have not been severe enough before to start medication. This part of the population may not be fully representative for the whole population. However, we do believe the exclusion criterion “current or previous use of methylphenidate in the past year” should be maintained, as medication-naïve children and those who have tried it in the past are less likely to have knowledge about the effects of medication, and are therefore more neutral in their expectations before starting medication, then children who have recently used or are currently using medication. We believe that the latter group of children already has a very positive or negative attitude towards medication, which may lead to expectation bias [113].

To conclude, this study will be the first to reveal whether mindfulness treatment for children with ADHD can be provided as an alternative to the current standard treatment of medication, and may reveal which mechanisms of change contribute to the effectiveness of the interventions. This study has the potential to impact clinical practice in important ways, as at present most children referred with ADHD receive medication, despite its disadvantages.

## Abbreviations

ADHD, Attention-Deficit-Hyperactivity-Disorder; ASR, Adult Self Report; CAMM, Children’s Acceptance and Mindfulness Measure; CBCL, Child Behavior Checklist; CSRQ, Chronic Sleep Reduction Questionnaire; DBDRS, Disruptive Behavior Disorder Rating Scale; DSM, Diagnostic and Statistical Manual of Mental Disorders; EDT, Emotion Discussion Task; FFMQ, 5 Facet Mindfulness Questionnaire; HSDQ, Holland Sleep Disorders Questionnaire; HSR, Healthy Self-Regulation Subscale; ICER, Incremental Cost-Effectiveness Ratios; IM-P, Interpersonal Mindfulness in Parenting scale; MBCT, Mindfulness Based Cognitive Therapy; MBSR, Mindfulness Based Stress Reduction; MM, Mind Mindedness; MTA, Multimodal Treatment of Attention Deficit Hyperactivity Disorder; NWO, Netherlands Organisation for Scientific Research; PS, Parenting Scale; PSOC, Parenting Sense of Competence; PSQI, Pittsburgh Sleep Quality Index; PSS, Perceived Stress Scale; QUALY, Quality Adjusted Life Year; RCT, randomized controlled trial; SCID, Structured Clinical Interview for DSM; SCS, Self Compassion Scale; SHS, Subjective Happiness Scale; SQC, Stress Questionnaire for Children; SQIH, Self-report Questionnaire for Inattention and Hyperactivity; TEA-Ch, Test of Everyday Attention for Children; TOF, Test Observation Form; TRF, Teacher’s Report Form; WHO-5, World Health Organization Well-Being Index; WISC, Wechsler Intelligence Scale for Children – Third edition; YSR, Youth Self Report.
